# The role of *Clostridium difficile* in the paediatric and neonatal gut — a narrative review

**DOI:** 10.1007/s10096-016-2639-3

**Published:** 2016-04-23

**Authors:** E. A. Lees, F. Miyajima, M. Pirmohamed, E. D. Carrol

**Affiliations:** University of Liverpool Institute of Translational Medicine, Wolfson Centre, Block A: Waterhouse Building, 1-5 Brownlow Street, Liverpool, L69 3GL UK; Department of Clinical Infection, Microbiology and Immunology, Institute of Infection and Global Health, Ronald Ross Building, West Derby Street, Liverpool, L69 7BE UK

## Abstract

*Clostridium difficile* is an important nosocomial pathogen in adults. Its significance in children is less well defined, but cases of *C. difficile* infection (CDI) appear to be increasingly prevalent in paediatric patients. This review aims to summarize reported *Clostridium difficile* carriage rates across children of different age groups, appraise the relationship between CDI and factors such as method of delivery, type of infant feed, antibiotic use, and co-morbidities, and review factors affecting the gut microbiome in children and the host immune response to *C. difficile*. Searches of PubMed and Google Scholar using the terms ‘*Clostridium difficile* neonates’ and ‘*Clostridium difficile* children’ were completed, and reference lists of retrieved publications screened for further papers. In total, 88 papers containing relevant data were included. There was large inter-study variation in reported *C. difficile* carriage rates. There was an association between CDI and recent antibiotic use, and co-morbidities such as immunosuppression and inflammatory bowel disease. *C. difficile* was also found in stools of children with diarrhoea attributed to other pathogens (e.g. rotavirus). The role of *C. difficile* in the paediatric gut remains unclear; is it an innocent bystander in diarrhoeal disease caused by other organisms, or a pathogen causing subclinical to severe symptoms? Further investigation of the development of serological and local host response to *C. difficile* carriage may shed new light on disease mechanisms. Work is underway on defining a framework for diagnosis and management of paediatric CDI.

**Electronic supplementary material:**

The online version of this article (doi:10.1007/s10096-016-2639-3) contains supplementary material, which is available to authorized users.

## Background

*Clostridium difficile* (*C. difficile*) is a Gram-positive, anaerobic spore-forming bacillus, which can exist as both toxigenic and non-toxigenic forms [1]. It has become a significant cause of nosocomial infection with high mortality rates, particularly in the elderly. There is increasing interest in the changing epidemiology of *C. difficile*, as mortality rates have risen in association with emergence of hypervirulent strains such as the toxinotype group V, PCR ribotype 078 (NAP7/BK/078) and North American toxinotype III, PCR ribotype 027 (NAP1/BI/027), and there have been increasing rates of community-associated disease in recent years [2]. Approximately 4–5 % of non-hospitalised healthy adults carry the organism in their intestinal flora [3]. In hospitalised adults and those in long-term care facilities, the rate of asymptomatic carriage is estimated to be 20–50 % [4, 5], and varying carriage rates of up to 70 % have been reported in healthy newborns [6]. In children, there is a decreasing trend in carriage rate with increasing age; with colonisation falling to adult levels of around 5 % by the age of 2 years.

*C. difficile* colonisation results in a spectrum of clinical conditions ranging from asymptomatic carrier state to fulminant colitis. The pathophysiology of *C. difficile*-associated diarrhoea requires alteration of the colonic microflora, colonisation by *C. difficile*, and the release of enterotoxins from the toxigenic strains (typically toxin A and toxin B and in some instances a binary toxin) [1]. The use of broad-spectrum antibiotics disturbs the indigenous intestinal microbiota, which eliminates competing microbes and allows *C. difficile* overgrowth and toxin production in the colon.

Researchers have tried to identify the differences in host mechanism between adult and paediatric populations, as *C. difficile* has traditionally been viewed as non-pathogenic in young infants, given that they may carry both toxigenic and non-toxigenic strains without overt clinical symptoms. One theory is that infants lack the mechanism for cellular internalization of the large clostridial toxins owing to their presumed lack of toxin receptors, which purportedly reach adult levels after weaning [7]. Some studies have considered the protective mechanisms of breast milk in *C. difficile* colonisation in comparison to artificial formula [8, 9]. An in-vitro and in-vivo study showed that human colostrum contains neutralizing antibodies to toxins A and B [6, 10]. A study examining the association between serum IgG antitoxin A levels and development of clinical symptoms found that adults with low or absent antibody levels were more likely to develop diarrhoea or colitis, whereas those with higher titres were more likely to exhibit asymptomatic carriage [11]. Similarly, relapse/recurrence of CDI occurred more frequently in individuals with lower levels of IgG/IgM to Toxin A [12], but there are no reported data on when infants develop seropositivity to *C. difficile* antigens, and whether this correlates with the clearing of the organism from the bowel flora or with symptomatic *C. difficile* infection.

Concern about *C. difficile* disease in children has resurfaced due to the higher rates of infections and recurrence found in specific groups of children, such as children with haematological malignancies, inflammatory bowel disease (IBD), and cystic fibrosis following lung transplantation [13]. Although there have been a number of epidemiological studies performed in the United States [14] and Canada, large gaps in our knowledge remain as to the role of *C. difficile* and its interaction with other bowel flora in neonates and children. There is also controversy over whom to test for *C. difficile*, with the American Academy of Pediatrics releasing a policy statement in 2013 outlining when *C. difficile* testing should be considered in children — recommending avoidance of routine testing in children under 1 year of age, due to their higher carriage rates. Between 1–3 years, testing may be considered, but testing for other pathogens (especially viral pathogens) should be prioritized. Over 3 years, it is advised that testing should be performed in the same circumstances as it would be in adults (i.e., acute diarrhoea and recent history of antibiotic use) [14].

First-line treatments for *C. difficile* disease are vancomycin or metronidazole, although in 22–38 % of cases (particularly in severe disease), failure of treatment has been reported with metronidazole. Disease relapse/recurrence is also a concern with both drugs [15]. More recently, fidaxomicin, the first in a new class of macrocylic antimicrobials against *C. difficile*, has been introduced with greater efficacy in patients with recurrent disease, though data is lacking in use for patients below 18 years of age [16, 17]. Pharmacokinetic study of the drug in children 6 months–18 years is underway in the USA (Clinicaltrials.gov: NCT01591863), but expert panel has suggested that there is no unmet need for a new treatment in children under 2 years, given the lack of a clear case definition in this population [18].

In recent years, with rapid advances in genetic sequencing techniques, there has been increasing interest in the human gut microbiome. The microbiome constitutes the many and varied microbes (including bacteria, viruses, archaea, and fungi) that colonize the skin, oral cavity, and gut shortly after birth in all humans [19]. These microbes are generally thought to be commensals; however, their particular composition is thought to play a role in certain illnesses (e.g., IBD) [20, 21]. There is an association between reduced diversity of the gut microbiome, intestinal dysbiosis, *C. difficile* carriage [22], and *C. difficile* disease/recurrence in adults [23]. Analysis of *C. difficile*-infected mice found that the microbiota consistently contained (in addition to *C. difficile*) opportunistic pathogens that have been identified within the microbiota of humans with CDI. These pathogens include: *Klebsiella pneumoniae*, *Proteus mirabilis*, and *Enterococcus faecalis* [22]. *Klebsiella pneumoniae* and *Ruminococcus gnavus* were noted to be associated with *C. difficile* carriage in an infant study, with *Bifidobacterium longum* appearing to have a protective role [24]. In addition, administration of targeted bacteriotherapy (with a mixture including *Lactobacillus reuteri* and *Bacteroidetes sp. nov.*) to mice with chronic CDI was able to eliminate disease and shedding by restoring a more diverse intestinal microbiota [22].

## Objectives

The objectives of this review are:

1. To summarise current available evidence on prevalence and distribution of *C. difficile* in neonates, infants, and children.
2. To ascertain the relationship between *C. difficile* infection (CDI) and factors such as delivery method, infant feed type, environmental exposure (e.g., time spent on NICU), antibiotic use, and co-morbidities.
3. To summarise risk factors for relapse of CDI, and review factors affecting the gut microbiome in children and the immunological response to *C. difficile* in childhood.

## Methods

### Search methods for identification of studies

#### Electronic searches

Searches of PubMed and Google Scholar were completed, using the terms ‘*Clostridium difficile* neonate/newborn/infant’ and ‘*Clostridium difficile* child/children’. The Cochrane Library and the Public Health England (formerly Health Protection Agency) websites were searched for current UK guidance on *C. difficile* infection. Reference lists of retrieved publications were screened for further papers. A total of 51 articles containing epidemiological data on *C. difficile* were reviewed and included (see appendix 1 and Supplement 1). Of these, 23 studies contained data for participants 0 to 1 month old, 18 for participants 1 month to 1 year old, and 26 studies contained data for participants >1 year of age. Reported studies were conducted between 1981 and 2013, in diverse locations worldwide and in both high- and low-resource settings. Sample size ranged from 12 to 1032 and a wide range of participants were involved, including: inpatients on Neonatal Intensive Care Unit (NICU) and post-natal wards, healthy outpatients, nursery attendees, hospitalised children with and without diarrhoea, and immunocompromised children. In addition to these studies, 37 articles were identified that also provided data relevant to issues addressed in objectives 2 and 3.

### Data collection and analysis

#### Selection of studies

Studies were included if they were written in English and offered data on *C. difficile* prevalence in children, regardless of setting (i.e., inpatient/outpatient/healthy volunteer). Results of epidemiological studies were split into three age groups: neonates < 1 month, infants 1 month to 1 year, and children >1 year of age. If the age range of patients was not specified, results were included in the category that contained the median and mean age (where recorded). If median and mean were not specified and the exact number of participants in each age group was not determined from the figures in each study, data were included in the group into which the greatest number of patients appeared to fall (this occurred for four studies, and these are highlighted in online supplement 1). The initial intention had been to determine rates specifically in the age group of children >2 years, to correspond with the current accepted lower age limit for *C. difficile* testing in most UK paediatric centres; however, few studies included data on this age group separately from those >1 year of age.

Given the heterogeneous nature of the study populations, and lack of measurable health outcomes (given that some studies assessed *C. difficile* prevalence, whereas others treated those with *C. difficile* as having disease), it was felt that a meta-analysis was not appropriate for these data. Data analysis was performed using STATA statistical software (release 14.0, STATA Corp., College Station, TX, USA) and Microsoft Excel.

## Results

### Carriage rates in different age groups

**Fig. 1:**
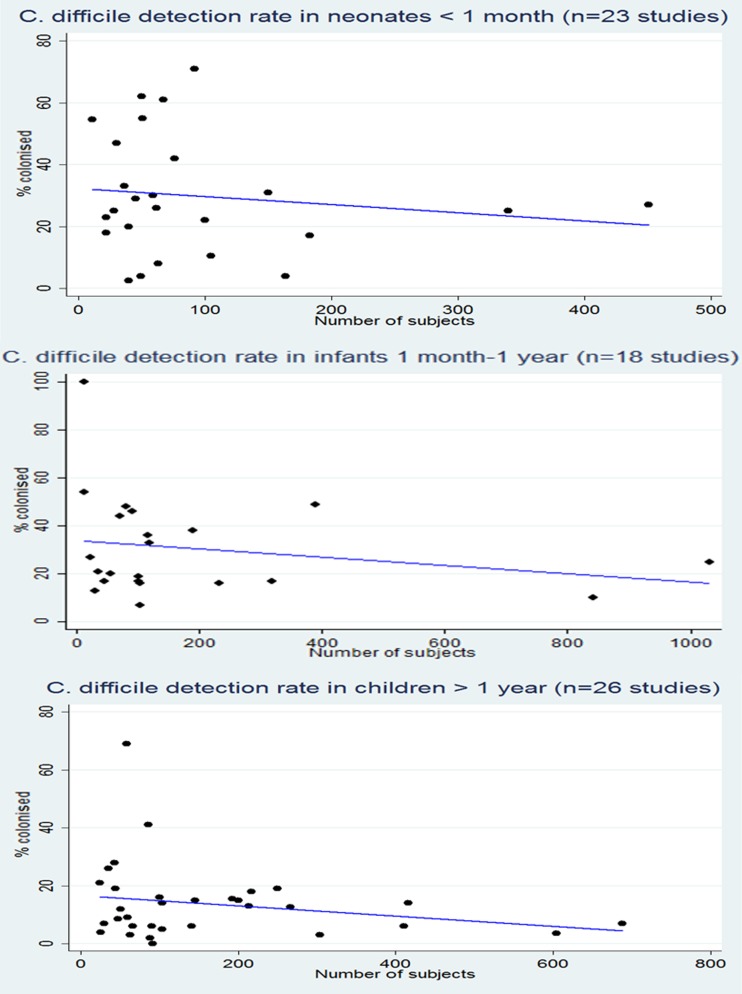
*C. difficile* detection rate in populations by age

**Fig. 2:**
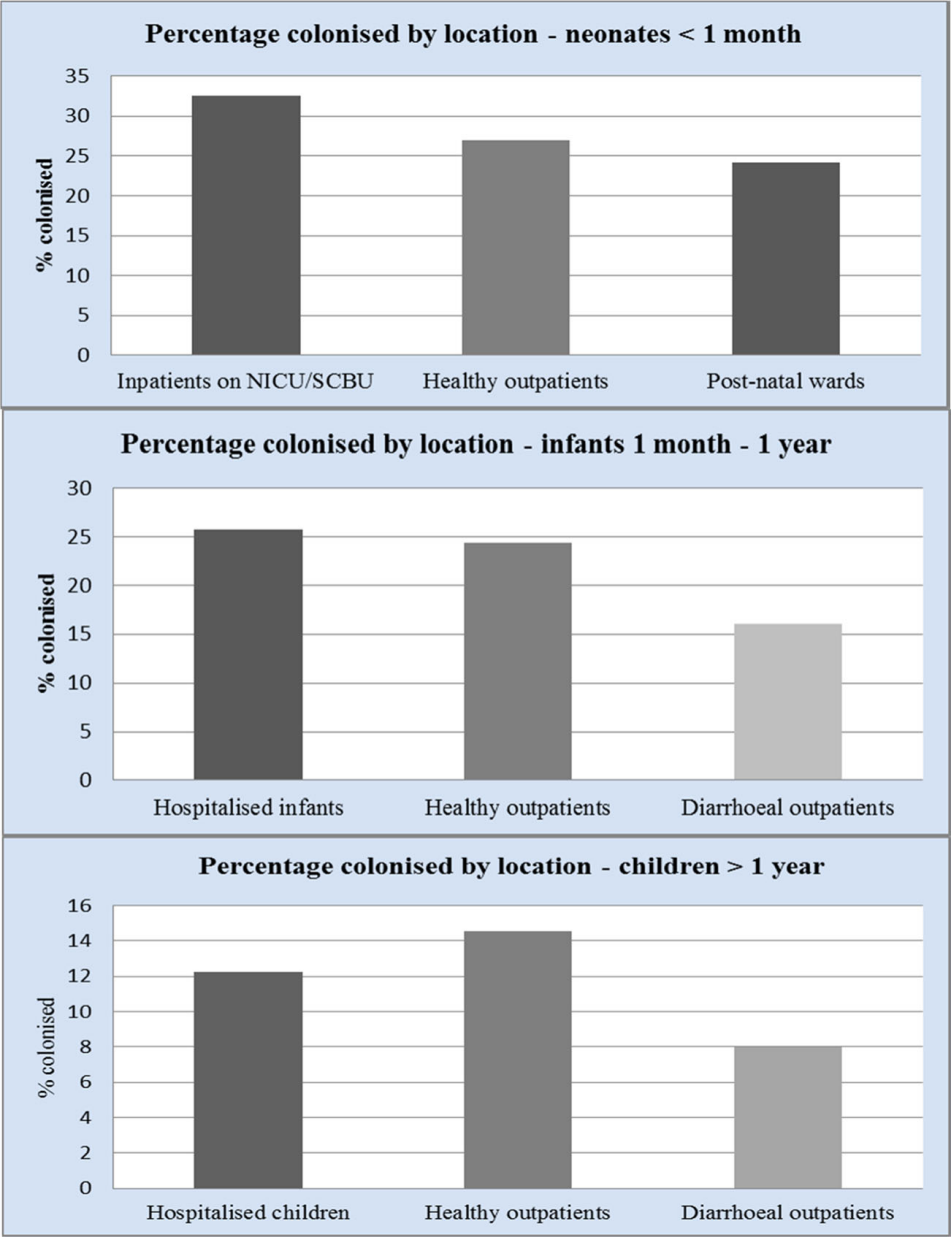
Percentage of subjects colonised by location

For each age group, there was a large disparity in rate of *C. difficile* colonisation (Fig. 1); a volume effect was seen in larger studies, suggesting rates of 25–30 % in neonates under 1 month of age, 10–25 % in infants 1 month to 1 year of age, and 5–10 % in children >1 year old; the latter value approaches the rate seen in adult studies. We also analysed data by location of patients tested; for neonates, the highest rates of colonisation were seen in patients on NICU (33 %), suggesting persistence of *C. difficile* in the NICU environment, but high rates were also seen in healthy outpatients (27 %) and on the postnatal ward (24 %) (Fig. 2).

For infants 1 month to 1 year of age and those over 1 year of age, comparable rates of *C. difficile* were seen in hospitalised infants (26 % and 12 % respectively) and healthy outpatients (24 % and 15 % respectively). Interestingly, *C. difficile* was isolated least frequently in diarrhoeal outpatients (16 % and 7 % respectively).

### Association between environment, antibiotic use, infant feed type, delivery method, and colonisation in neonates

#### Environmental exposure

Rates of *C. difficile* detection have been shown to increase with length of stay on the neonatal unit; some studies have also shown clear patterns of environmental contamination [25, 26]. Kato et al. reported a 61 % colonisation rate of infants in a NICU, with 53 of 55 isolates from 30 patients being identical and non-toxigenic, suggesting nosocomial spread on the NICU [27], whereas Merida et al. found no carriage of *C. difficile* in term neonates born on a recently opened maternity unit, where it is likely that environmental contamination had not yet occurred [28].

#### Antibiotic use

In a previous US study, antibiotic treatment for neonates on NICU was associated with a lower *C. difficile* colonisation rate [29], but colonisation with *C. difficile* occurred rapidly after cessation of antibiotics. The presence of *C. difficile* colonisation and other faecal microflora is delayed in infants who receive antibiotic treatment [30, 31]. There was an inverse correlation between gut diversity index (number and distribution of bacterial species in the gut) and number of days of antibiotics received in infants born between 27 and 29 weeks’ gestation, and weight gain increased with increased diversity scores [32].

None of the neonates in the studies reviewed were treated for *C. difficile*, with the exception of the study by Han et al. [33], where neonates admitted to NICU who were found to carry *C. difficile* were treated with vancomycin due to an outbreak of necrotizing enterocolitis (NEC) attributed to *C. difficile*. However, since other bacterial gut pathogens were not investigated, a causal link here between *C. difficile* and NEC cannot be inferred.

#### Method of infant feeding

Penders et al. [34], found that in healthy 1-month-old infants who had not received antibiotics, twice as many formula-fed infants were colonised by *C. difficile* than those who were exclusively breast fed. Also, those breast-fed infants who were colonised by *C. difficile* had significantly lower colony counts than formula-fed infants. These findings were replicated with a study of infants up to 1 year of age, demonstrating colonisation rates of *C. difficile* to be 4 times higher in formula-fed infants than in those who were exclusively breast-fed. Those receiving both breast and formula feeds had intermediate colonisation rates [8]. A large Swedish study also found significantly more formula-fed infants under 6 months to be colonised with *C. difficile* [9]. Ruminococcus (which is more commonly found in the gut of breast-fed infants), is thought to inhibit growth of Clostridia, thereby preventing colonisation by *C. difficile* [35].

#### Mode of delivery

A small UK study found 22 % women had vaginal colonisation by *C. difficile* either pre- or post-delivery; of these, 89 % delivered infants whose stools tested positive for *C. difficile* within 4 days of birth, compared to a 56 % detection rate in infants born to swab-negative mothers [36]. These findings have not been replicated in other maternal studies.

A delay in colonisation and alteration in composition of microbiota (with lower counts of *Bifidobacteria*) has been noted in babies delivered by caesarean section (CS) relative to those born by vaginal delivery [37, 38]. Another study found lower bacterial counts until day 7 of life in infants delivered by planned caesarean (i.e., amniotic membranes intact and no exposure to vaginal/bowel flora) [39]. This study, which sampled neonatal stools regularly over the period of 1 year, also demonstrated that whilst the broad groups of bacteria found in the GI tract were similar for each participant, there was a great degree of diversity and individuality in the combination of species that each infant acquired and their change over time. The microbiome was more stable than anticipated, even in the neonatal period, with certain colonizing bacteria being found in the stool repeatedly over a period of weeks or months. Fraternal twins had similar microbiomes, indicating the important influence of environment on colonisation. By 1 year of age, the microbiome was starting to resemble an adult profile, though interestingly, there were no great similarities between infants and their own parents.

A longitudinal study of term infants born by CS (with prophylactic antibiotics) or vaginal delivery noted significantly lower bacterial counts in the CS group, even at 6 months of age and delayed colonisation by *Bacteroides*. They were also significantly more likely to be colonised by *Clostridium perfringens*. No differences up to this point were noted in intestinal signs and symptoms, such as diarrhoea or colic [40]. A study recruiting healthy Finnish children at 7 years of age found significantly higher numbers of Clostridia in the stool of children delivered vaginally than in those delivered by CS. This study suggests that method of delivery may have persistent effects on microbiota well beyond infancy [41].

### *C. difficile* burden in children over 1 year

In England, Wales, & N. Ireland in 2014, 257 cases of CDI were reported in children <15 years, representing a 5.9 % decrease on the previous year’s figures [42]. When analysed by age, rates of CDI were (expressed as cases per 100,000 population): <2 years = 2.7, 2–4 years = 4.1, 5–9 years = 1.8 and 10–14 year s = 2.0.

In a recent US-based study, the incidence of CDI in children had increased remarkably, from 2.6 to 32.6 per 100,000 person-years between the periods 1991–1997 and 2004–2009 [43]. Cases were defined as diarrhoea with positive C. *difficile* enzyme immunoassay (EIA), or PCR and no other identifiable cause for diarrhoea. There was a sharp increase in cases from 2006 to 2008, but the C*. difficile* detection method was altered from EIA to PCR in July 2007, suggesting increased case ascertainment with use of PCR. Of all cases, 75 % were community-attributable, but interestingly, 85.5 % of these patients had reported an ED or outpatient visit in the 3 months prior to disease onset.

A 2014 Polish study reports rates of 13.5 cases of CDI per 1,000 children hospitalised with diarrhoea; however, not all children were tested for viral/alternative bacterial pathogens, and those who were co-infected with *C. difficile* and another pathogen were recorded as being a case of CDI [44]. Indeed, a recent literature review on co-infections in children with C. difficile notes a rate of 20.7 % (range 0–100 %) for reported co-infections (predominantly viral infections), but found it difficult to draw any meaningful conclusions, given the heterogeneity between studies as to which organisms were tested for (virus/bacteria/parasite) and the difficulty in describing what constitutes CDI in the paediatric population [45].

Where data on treatment of cases was available for *C. difficile* positive subjects in the studies reviewed here of infants 1 month to 1 year or children >1 year, 23/968 cases (2.4 %) aged 1 month to 1 year and 86/368 (23.4 %) >1 year received treatment with metronidazole and/or vancomycin for their suspected CDI.

A recent study of adults in Oxfordshire (UK) noted a decrease in prevalence of the hypervirulent ST1/NAP/027 strain, which was proposed to be due to improved antibiotic stewardship and infection control measures. Interestingly, this study also found a fairly diverse reservoir of *C. difficile* subtypes (with 45 % of CDI cases being genetically distinct from one another), and a further 36 % patients with CDI from genetically similar strains having no hospital or community contact with one another [46]. This suggests the existence of other reservoirs for infection, possibly asymptomatically colonised infants. Contact with children under 2 years of age has also been linked with CDI in adults with community-acquired disease, with 14 % of cases vs 2 % of controls having reported contact with a child under 2 [47].

### Risk factors for *C. difficile* infection (CDI)

In the US, 57 % to 75 % of paediatric patients with community-acquired CDI reported antibiotic use in the 3 months prior to admission [48]. In children hospitalised with CDI, exposure to three antibiotic classes in the month prior to admission was associated with severe disease, as was malignancy [49]. No association was found with age, prematurity (delivery <37 weeks’ gestation), GI surgery, or steroid/immunosuppressant use for 2 weeks in the month preceding diagnosis. Other studies have shown a strong link between immunosuppression and severe illness with CDI [50, 51]. Inflammatory bowel disease (IBD) [52], intestinal stasis (Hirschsprung’s disease) [13], organ transplant and gastrostomy/jejunostomy and cystic fibrosis [53] have all been implicated in the development of CDI [48]. Indeed, the first presentation of IBD could appear clinically similar to that of *C. difficile* enterocolitis. A US study found a significantly increased incidence of *C. difficile* in children >3 years with previous antibiotic exposure (35 % exposed vs 2 % unexposed) [54]. Another study reported association between community-acquired CDI and cephalosporin use within 30 days (OR 3.32; 95 % CI: 1.10–10.01) and presence of a gastrointestinal feeding device (OR 2.59; 95 % CI: 1.07–6.30) [55].

Elevated gastric pH has been hypothesized to influence CDI risk by facilitating bacterial colonisation of the upper gastrointestinal tract and/or survival of the vegetative phase of *C. difficile* in the stomach. An Italian paediatric retrospective case–control study found a significant association between proton-pump inhibitor (PPI) usage and CDI (PPI use in 22.1 % *C. difficile*-positive vs 5.9 % *C. difficile*-negative patients) and a non-significant association between H2-receptor antagonists and CDI (10.3 % vs 2.9 %) [56].

There have also been reports of CDI associated with viral gastroenteritis (norovirus and caliciviruses), with a suggested mechanism of inflammation of the intestinal epithelium following gastroenteritis facilitating adherence and colonisation by *C. difficile* and the attachment of its toxins [57, 58]. In an Italian study, co-infection with multiple pathogens was seen in 27 of 151 patients (18 %), with the most common co-infections being rotavirus and toxin-producing *C. difficile*, accounting for 63 % of co-infections [59]. Children with co-infection had a higher incidence of severe disease and were more likely to be dehydrated on presentation. There was no significant age difference between those with and without co-infection. All cases of *C. difficile* occurred in children under 6 years, with the majority being concentrated in the youngest group (under 2 years). Conversely, another study noted that diarrhoeal children with viral co-infections tended to have higher *C. difficile* bacterial burden (median difference  =  565,957 cfu/ml; *p* =   0.011), but were clinically indistinguishable from those with *C. difficile* alone [60]. Similarly, an Italian study found no significant difference in clinical presentation in children with prolonged/mucohaemorrhagic diarrhoea by *C. difficile* status [61]. The significance of detecting *C. difficile* in the presence of known pathogenic viruses remains unclear.

### Relapsing infection in children

Relapses and/or recurrences have been reported in up to 25 % of paediatric CDI cases [62], which is in line with 20–30 % seen in adult studies [63]. In adults, relapse has been associated with lower blood concentrations of anti-toxin A and B antibody and with the presence of strain BI/NAP1/027 [11, 64]. This has led to use of pooled intravenous immunoglobulins (IVIg) and anti-toxin monoclonal antibodies in recurrent, refractory, or severe disease. There has been little work done in children, but a small study by Leung et al. reported lower serum levels of IgG for TcdA in children with relapsing *C. difficile*-associated colitis than in healthy children, with symptoms ameliorating following IVIg infusions [65]. There was, however, no control group in this study so it is difficult to infer causation, although a study of CDI in children found that transient hypogammaglobulinaemia of infancy was significantly associated with recurrent disease, adding credence to this theory [66].

The reported incidence of complications with CDI in children (e.g., renal failure, bowel perforation, death) is lower than that in adults, varying between 0–12 % [67]. However, it is difficult to classify *C. difficile* disease in children, as there is no validated paediatric tool and current guidance comes from the adult classification systems. One retrospective paediatric study found that although 76 % of the cases seen in their hospital would be classified as severe using adult guidelines, most cases experienced a fairly mild illness with low morbidity and mortality [68]. The majority of patients in this study had not been treated or were given probiotics or metronidazole with good recovery, despite their ‘severe’ classification. The authors proposed new disease classification criteria for children which are currently undergoing prospective validation.

### Faecal biomarkers and paediatric CDI

Faecal *C. difficile* bacterial load does not appear to differ between symptomatic and asymptomatic children and does not correlate with outcome [69]. In this study, faecal levels of lactoferrin and cytokines (CXCL-5, IL-8) were elevated in *C. difficile-*positive compared to *C. difficile-*negative children with diarrhoea, and time to diarrhoea resolution after treatment was significantly longer in those with elevated faecal CXCL-5 mRNA, and IL-8 mRNA at diagnosis (medians of 7 vs 2 days and 5 vs 3 days respectively). A relatively small sample size of 102 may limit further generalization from this study’s results. Similarly, immunosuppressed CDI patients had lower IL-8 mRNA expression than immunocompetent patients.

### Serological response to exposure to *C. difficile*

A study of infants under 6 months of age found that 11 % and 33 % had detectable levels of serum IgG against toxins A and B respectively. Prevalence of serum anti-toxin IgG to both toxins increased throughout childhood, reaching 25 % (toxin A) and 53 % (toxin B) by 2 years [70]. Likelihood of strongly reactive antibodies (as measured by ELISA values) also increased with age over 2 years. Interestingly, those who produced a strong antibody response against toxin A were less likely to produce an equivalent response against toxin B and vice versa.

## Conclusions

It is accepted that *C. difficile* is present relatively frequently in neonates, though its significance and effects on the microbiota in later life have yet to be determined. Possible hypotheses for lack of *C. difficile* disease in this population include: immaturity of bowel mucosa with a lack of receptors for *C. difficile* toxins, immunoglobulin fractions present in breast milk preventing binding of toxins to their receptors, as well as the nature and composition of infant gut microflora being protective against *C. difficile* overgrowth [71]. Further studies are needed to determine the significance of asymptomatic *C. difficile* colonisation and consequent changes in the microbiota throughout infancy and childhood and into later life.

There is a huge body of literature on *C. difficile* infection in adults, and now an expanding body of work on its role in children. There remains a great deal of disagreement on what constitutes paediatric *C. difficile* infection, and the differentiation between symptomatic manifestation and what is believed to be presence of the organism as a bystander in diarrheal disease caused by other organisms. A collaborative policy document published by the Society for Healthcare Epidemiology of America supports the view that in the setting of high prevalence of asymptomatic carriage, *C. difficile* cannot be assumed to be the causative agent of diarrhoea prior to adolescence (particularly in younger children) [72].

Defining paediatric CDI is further complicated by the lack of a standardized scoring system for paediatric infection, making it more difficult to quantify disease burden in those thought to have CDI and thus to know whom to treat. Crews et al. [55] suggest a framework as to how severity of disease may be defined in children, and clearly consideration of the presence of risk factors should play an additional role in ascertaining the likelihood of CDI versus incidental finding. Given the consensus that children who do have CDI run a much milder disease course than adults, it is appropriate to tailor treatment as such, with the first steps being supportive care (rehydration) and discontinuation of unnecessary antibiotics, or at least narrowing spectrum and reviewing course length, prior to considering active treatment with metronidazole/vancomycin.

Longitudinal exploration of the role of the intestinal microbiota and the development of serological host response to *C. difficile* during carriage and disease and the age at which this occurs would prove valuable approaches to this issue. This, alongside more detailed work on local gut response to *C. difficile* in diarrheal children, would provide a firm basis for the mechanistic understanding of pathogenesis of CDI in early life. Further work is also warranted on the hypothesis that children are a major community reservoir for community-attributable CDI cases in adults, as this would have important public health implications.

### Electronic supplementary material

Below is the link to the electronic supplementary material.

Supplement 1 (PDF 664 kb)
