# Supplementary material for: The role of Clostridium difficile in the paediatric and neonatal gut — a narrative review
Source: Eur J Clin Microbiol Infect Dis. 2016 Apr 23;35:1047–57. doi: 10.1007/s10096-016-2639-3 (PMC4902830; doi:10.1007/s10096-016-2639-3)
Supplement: Supplementary file 1 — (PDF 664 kb) [file 10096_2016_2639_MOESM1_ESM.pdf]

Supplement 1: Description of studies included in epidemiological analysis

Year	Location	Number of subjects	Study setting	Frequency of colonisation	Authors
**Neonates < 1/12**					
2012	France	76	Inpatients on NICU	42%	Ferraris et al[73]
2011	France	62	Healthy outpatients/ hospitalised infants	26%	Rousseau et al[74]
2005	Netherlands	100	Healthy outpatients	22%	Penders et al[34]
2005	Japan	22	Healthy outpatients	18%	Tonooka et al[75]
2005	Japan	40	Post natal wards	2.5%	Matsuki et al[76]
1995	Poland	183	Post natal wards	17%	Martirosian et al[77]
1994	Japan	67	Inpatients on NICU	61%	Kato et al[27]
1989	Sweden	340	Post natal wards	22-28%	Tullus et al[9]
1986	Sweden	164	Inpatients on NICU	4%	Bennet et al[38]
1986	Belgium	63	Hospitalised infants	8%	Merida et al[28]
1985	Nigeria	22	Inpatients on NICU	23%	Rotimi et al[78]
1984	UK	150	Post natal wards/ healthy outpatients	31%	Bolton et al[79]
1984	UK	50	Post natal wards	62%	Tabaqchali et al[36]
1984	UK	92	Inpatients on NICU	71%	Al-Jumaili et al[80]
1983	Canada	30	Inpatients on NICU	47%	Han et al[33]
1983	UK	11	Inpatients on NICU	54.5%	Malamou-Ladas et al[81]
1983	UK	40	Post natal wards	20%	Malamou-Ladas et al[81]
1983	UK	59	Post natal wards	30%	Richardson et al[82]
1983	USA	36	Healthy outpatients	33%	Cooperstock et al[8]
1982	UK	451	Post natal wards	2-52%	Larson et al[25]
1982	USA	51	Inpatients on NICU	55%	Donta[29]
1982	USA	105	Post natal wards	10.5%	Donta[29]
1982	Australia	28	Inpatients on NICU	25%	Blakey et al[30]
1981	USA	45	Healthy outpatients	29%	Viscidi et al[83]
1981	Sweden	49	Diarrhoeal inpatients/ outpatients/ Nursery attendees	4%	Holst et al[84]
**Infants 1/12 – 1yr**					
2011	France	189	Healthy outpatients/ hospitalised infants	38%	Rousseau et al[74]
2009	Iraq	46	Nosocomial diarrhoea	17%	Alrifai et al[85]
2006	Netherlands	1032	Healthy outpatients	25%	Penders et al[37]
2005	Japan	12	Nursery attendees	100%	Matsuki et al[76]
2005	USA	104	Diarrhoeal outpatients	16%	Denno et al[86]
2005	Canada	841	Diarrhoeal inpatients	10%	Tang et al[87]
1997	Sweden/Estonia	56	Healthy outpatients	20%	Naaber et al[88]
1997	Sweden/Estonia	34	Hospitalised infants	21%	Naaber et al[88]
1989	Sweden	307-331	Healthy outpatients	10-28%	Tullus et al[9]
1989	Korea	104	Healthy outpatients	7%	Kim et al[89]
1989	Korea	231	Diarrhoeal	16%	Kim et al[89]

			outpatients		
1989	USA	30∞	Nosocomial diarrhoea	13%	Brady et al[90]
1986	Belgium	91	Hospitalised infants	46%	Merida et al[28]
1984	Mexico	100	Hospitalised infants	19%	Torres et al[91]
1984	Mexico	22	Nursery attendees	27%	Torres et al[91]
1984	UK	390*	Diarrhoeal inpatients	49%	Ellis et al[92]
1984	UK	118*	Non-diarrhoeal inpatients	33%	Ellis et al[92]
1983	USA	71	Healthy outpatients	44%	Cooperstock et al[8]
1982	Australia	13	Healthy outpatients	54%	Stark et al[93]
1982	UK	116§	Diarrhoeal inpatients/ outpatients	36%	Nash et al[94]
1982	Sweden	100^	Healthy outpatients	17%	Svedhem at al[95]
1981	Sweden	81	Diarrhoeal inpatients/ outpatients/ Nursery attendees	48%	Holst et al[84]
**Children > 1yr**					
2013	USA	51	Patients with non-inflammatory bowel conditions	12%	Lamouse-Smith et al[96]
2013	USA	145	Patients with IBD	15%	Lamouse-Smith et al[96]
2011	France	43	Healthy outpatients/ hospitalised infants	28%	Rousseau et al[74]
2010	Poland	58	Patients with IBD	69%	Banaszkiewicz et al[97]
2009	Iraq	35	Nosocomial diarrhoea	26%	Alrifai et al[85]
2009	Italy	141	Oncology inpatients	6%	Castagnola et al[98]
2008	Germany	411	Oncology inpatients	6%	Simon et al[99]
2006	USA	688	Diarrhoeal outpatients	7%	Klein et al[54]
2006	USA	604	Healthy outpatients	3.5%	Vernacchio et al[100]
2005	India	250	Nosocomial diarrhoea	19%	Gogate et al[101]
2005	Japan	86	Nursery attendees	41%	Matsuki et al[76]
2005	USA	104	Diarrhoeal outpatients	5%	Denno et al[86]
2004	Egypt	104	Oncology inpatients	14%	El-Mahallawy et al[51]
2003	Brazil	91	Healthy outpatients	0%	Ferreira et al[102]
2003	Brazil	90	Diarrhoeal outpatients	6%	Ferreira et al[102]
2003	Brazil	63	Healthy outpatients	3%	Pinto et al[103]
2003	Brazil	51	Diarrhoeal outpatients	12%	Pinto et al[103]
2003	Brazil	30	Diarrhoeal inpatients	7%	Pinto et al[103]
2003	Brazil	66	Inpatients on antibiotics/ neutropaenic	6%	Pinto et al[103]
2002	Canada	217	Nosocomial diarrhoea	18%	Langley et al[104]
2001	Turkey	100	Nosocomial diarrhoea	16%	Oguz et al[105]
1998	USA	267	Diarrhoeal inpatients	13%	Shastri et al[106]
1997	Italy	193	Diarrhoeal inpatients (with IBD)	15.5%	Pascarella et al[52]
1997	Australia	60	Diarrhoeal oncology inpatients	9%	Burgner et al[107]

1997	Australia	44	Non-diarrhoeal oncology inpatients	19%	Burgner et al[107]
1995	UK	214	Diarrhoeal oncology inpatients	13%	Schuller et al[108]
1989	USA	24	Nosocomial diarrhoea	21%	Brady et al[90]
1989	Sweden	304	Healthy outpatients	3%	Tullus et al[9]
1986	Belgium	25	Hospitalised children	4%	Merida et al[28]
1986	Sweden	200	Healthy outpatients	15%	Uhnoo et al[109]
1986	Sweden	416	Diarrhoeal inpatients/ outpatients	14%	Uhnoo et al[109]
1982	Australia	47	Healthy outpatients	8.5%	Stark et al[93]
1981	Sweden	88	Diarrhoeal inpatients/ outpatients/Nursery attendees	2%	Holst et al[84]

^Some participants 1 week-1 month but number not specified ∞Some participants 1-2 years but number not specified
*Some participants > 1 year but number not specified §Some participants 1-2 years but number not specified

Supplement to:

The role of *Clostridium difficile* in the paediatric and neonatal gut – a narrative review

Lees EA[1], Miyajima F[1], Pirmohamed M[1], Carrol ED[2]

Affiliations:
[1]University of Liverpool Institute of Translational Medicine, Wolfson Centre, Block A: Waterhouse Building, 1-5 Brownlow Street, Liverpool L69 3GL, United Kingdom
[2]Department of Clinical Infection, Microbiology and Immunology, Institute of Infection and Global Health, Ronald Ross Building, West Derby Street, Liverpool L69 7BE, United Kingdom

Contact details for corresponding author: emilylees@doctors.org.uk

Submitted to: EJCMID
